# VO_2_ as a Highly Efficient Electrocatalyst for the Oxygen Evolution Reaction

**DOI:** 10.3390/nano12060939

**Published:** 2022-03-12

**Authors:** Yun-Hyuk Choi

**Affiliations:** School of Advanced Materials and Chemical Engineering, Daegu Catholic University, Gyeongsan 38430, Korea; yunhyukchoi@cu.ac.kr

**Keywords:** electrocatalysis, oxygen evolution reaction, water splitting, vanadium oxide, nanoparticles

## Abstract

Herein, we report high electrocatalytic activity of monoclinic VO_2_ (M1 phase) for the oxygen evolution reaction (OER) for the first time. The single-phase VO_2_ (M1) nanoparticles are prepared in the form of uniformly covering the surface of individual carbon fibers constituting a carbon fiber paper (CFP). The VO_2_ nanoparticles reveal the metal-insulator phase transition at *ca.* 65 °C (heating) and 62 °C (cooling) with low thermal hysteresis, indicating a high concentration of structural defect which is considered a grain boundary among VO_2_ nanoparticles with some particle coalescence. Consequently, the VO_2_/CFP shows a high electrocatalytic OER activity with the lowest η_10_ (350 mV) and Tafel slope (46 mV/dec) values in a 1 M aqueous solution of KOH as compared to those of the vacuum annealed V_2_O_5_ and the hydrothermally grown VO_2_ (M1), α-V_2_O_5_, and γ′-V_2_O_5_. The catalytically active site is considered V^4+^ components and V^4+/5+^ redox couples in VO_2_. The oxidation state of V^4+^ is revealed to be more favorable to the OER catalysis compared to that of V^5+^ in vanadium oxide through comparative studies. Furthermore, the amount of V^5+^ component is found to be increased on the surface of VO_2_ catalyst during the OER, giving rise to the performance degradation. This work suggests V^4+^ and its redox couple as a novel active component for the OER in metal-oxide electrocatalysts.

## 1. Introduction

Renewable energy sources, such as wind and solar power, are providing an increasing share of the energy supply [1,2]. In particular, electrocatalytic water splitting is considered a promising strategy for the sustainable production of hydrogen from renewable energy sources [3,4,5,6,7]. However, overall water splitting suffers from the sluggish kinetics of anodic oxygen evolution reaction (OER) involving the four-electron oxidative half-reaction [8,9]. Therefore, an appropriate catalyst is necessary to accelerate the OER at low overpotentials (η) for enhanced energy conversion efficiencies [5]. The state-of-the-art OER catalysts are based on precious metals such as IrO_2_ and RuO_2_ with η close to 300 mV in a 1 M aqueous solution of KOH. However, the scarcity and high costs of those materials restrict their large-scale application [9]. In this regard, the earth-abundant transition-metal oxides based on Mn, Fe, Co, and Ni have been extensively investigated as alternative OER catalysts. In particular, Ni or Co-based layered double hydroxides (LDHs) exhibited high OER activities due to the oxidation or stabilization of Ni or Co ions forming the OER-active redox couples with the addition of Fe ion [8,10,11,12]. Also, spinel-type oxides such as Co_3_O_4_ and NiCo_2_O_4_ and amorphous Co-based oxides were proposed as promising OER catalysts [8,9,13,14]. Meanwhile, Ba_0.5_Sr_0.5_Co_0.8_Fe_0.2_O_3−δ_ (BSCF) perovskite was identified as the most promising OER catalyst based on orbital principles with the e_g_ occupation as a superior descriptor for oxygen evolution activity [15]. Other descriptor approaches of M-OH bond strength and (∆G_O_* − ∆G_HO_*) were suggested to find superior catalysts [16,17]. However, application of such descriptors is limited to experimental work and finding novel catalysts based on other transition metals such as Nb, Ta, and V [9,18]. Among transition metal oxides, OER activities of vanadium-based catalysts have seldom been explored although high OER activities for VOOH and Co-incorporated catalysts (CoVO_x_, Co_2_V_2_O_7_, Co_3_V_2_O_8_, and Co_3_V_2_O_8_) were recently highlighted [9,18,19,20]. However, we focused a spotlight on the fact that vanadium oxides (primarily, V_2_O_5_ and V_6_O_13_) exhibit excellent performance in various electrochemical devices such as Li-ion batteries and supercapacitors based on multiple vanadium oxidation states (V^2+^, V^3+^, V^4+^, V^5+^) and their redox couples [21,22,23,24,25,26]. Furthermore, the electrochemical reactivity and battery characteristics in VO_2_ have often been reported [27,28,29,30]. Oxidative desulfurization of dibenzothiophene for monoclinic VO_2_ was also reported [31]. V_2_O_5_ deposited on a high surface area oxide support (e.g., TiO_2_ and Al_2_O_3_) is widely known as effective catalysts for selective oxidation of sulfur dioxide, naphthalene, *o*-xylene, alkyl pyridine, chlorinated hydrocarbon, and mercury in the chemical, petroleum, and environmental industries [32]. Despite such investigations, OER activities of binary VO_2_ and V_2_O_5_ are rarely known.

In this work, we demonstrate a vacuum annealing approach for the direct integration of VO_2_ nanoparticles onto conductive carbon finer paper (CFP), making the integration uniform and highly adhesive at the centimeter scale. The use of CFP is also intended to increase the exposure of catalytically active sites of VO_2_ nanoparticles [33]. Since, in particular, VO_2_ undergoes a phase transition between insulating monoclinic phase (M1) and metallic rutile phase (R) at *ca.* 67 °C in the bulk [34,35,36,37], the structural characterization of the VO_2_/CFP involving the phase transition is carefully carried out. As a result, superior OER activity of VO_2_ with low η and Tafel slope values is revealed as compared to that of V_2_O_5_. This result can be a cornerstone for understanding the V^4+^-related active sites for OER and for the design of novel VO_2_-based OER catalysts.

## 2. Experimental

***Preparation of VO_2_ (M1) nanoparticles by vacuum annealing:*** To prepare VO_2_ nanoparticles coated on a carbon fiber paper (CFP), 0.1 g of V_2_O_5_ particles (Sigma-Aldrich, St. Louis, MI, USA) was dispersed in 10 mL of ethanol, followed by ultrasonication for 1 h. A solution loading of 100 μL/cm^2^ using a micropipette was cast onto a bare CFP (Toray Paper 120) substrate with dimensions of 2 cm × 1 cm size, which was cleaned by ultrasonication in acetone, deionized water, and ethanol. The solution-coated CFP was allowed to dry in air overnight and placed within an alumina boat, which was placed at the center of a 1-inch-diameter horizontal cold-wall quartz tube furnace equipped with a rotary pump system. For annealing, the tube furnace was heated to 950 °C at a ramp rate of 20 °C/min under vacuum condition (~10 mTorr). After holding at 950 °C for 10 min, the furnace was allowed to cool naturally to room temperature. The annealed coat on CFP was then removed from the center of the furnace for characterization and electrocatalytic evaluation. To prepare V_2_O_5_ nanoparticles coated on CFP, the same V_2_O_5_-dispersion solution was cast onto a bare CFP substrate in the same way and allowed to dry in air overnight. Then, the solution-coated CFP was placed within the alumina boat placed at the furnace center, followed by annealing. The reactor was heated to a temperature of 950 °C at a ramp rate of 20 °C/min under an O_2_ flow of 10 sccm and continuous vacuum pumping condition. After the temperature was held at 950 °C for 10 min, the furnace was then naturally cooled to room temperature.

***Preparation ofVO_2_ (M1) nanoparticles for comparison:*** VO_2_ (M1) nanoparticles were synthesized hydrothermally by adding 300 mg of V_2_O_5_ powder (Sigma-Aldrich) and 450 mg of oxalic acid (Fisher Scientific, Waltham, MA, USA) to 16 mL of deionized water (ρ = 18.2 MΩ/cm, purified using a Barnstead International NANOpure Diamond system) in a 23 mL polytetrafluoroethylene cup, as reported in previous work [38]. The reaction mixture was heated within an autoclave to 250 °C for 72 h. A matte-black powder was collected by vacuum filtration and washed with copious amounts of acetone and deionized water.

***Preparation ofα-V_2_O_5_ nanowires for comparison:*** α-V_2_O_5_ nanowires (with the average diameters of between 150–250 nm and lengths ranging from 1–100 μm) were synthesized according to a previously reported method [39]. In a typical reaction, 1.6 g of V_2_O_5_ (Beantown Chemical, Hudson, NH, USA, 99.5%) were added to a 125 mL capacity Teflon-lined autoclave. Subsequently, 80 mL of deionized water (ρ = 18.2 MΩ/cm) and 8 mL of 2-propanol (ACS reagent grade) were added. The autoclave was heated at 210 °C for 48 h. Following the heating period, the autoclave was removed from the oven and allowed to cool autogenously. The resulting green to blue powder (V_3_O_7_·H_2_O) was separated by filtration and washed with copious amounts of water and 2-propanol and allowed to dry in air overnight. The dried paper-like product was placed in a ceramic crucible and heated in open air in a muffle furnace at 350 °C for 72 h with stirring every 24 h. The resulting yellow-orange paper-like product was lightly ground and used in further experiments without any subsequent modification.

***Preparation ofγ′-V_2_O_5_ microrods for comparison:*** The metastable γ′-V_2_O_5_ polymorph microrods (with average widths of 0.9–1.1 μm) were synthesized according to a previously reported synthesis [40]. In a typical reaction, about 1 g of α-V_2_O_5_ was added to 40 mL of acetonitrile (MilliporeSigma, Burlington, MA, USA, <50 ppm H_2_O, 99.8%) under inert atmosphere. Subsequently, 1.25 molar equivalents (typically *ca.* 1 g) of LiI (Alfa Aesar, Haverhill, MA, USA, anhydrous, 98%) were added to the suspension. The reaction was allowed to proceed without stirring under inert atmosphere for 72 h. The dark green-blue γ-LiV_2_O_5_ product was separated by vacuum filtration and washed with copious amounts of acetonitrile and 2-propanol and allowed to dry in air overnight. The γ-LiV_2_O_5_ powder was then dispersed in 40 mL of acetonitrile under an inert atmosphere. Subsequently, 1.25 molar equivalents of NOBF_4_ were added to the suspension, resulting in the complete topotactic deintercalation of lithium from the structure. The resulting orange/red powder was separated from the suspension by vacuum filtration, washed with copious amounts of acetonitrile and 2-propanol, and used without further modification.

Each 0.1 g of the prepared VO_2_ (M1), α-V_2_O_5_, and γ′-V_2_O_5_ products was dispersed in 10 mL of ethanol, followed by ultrasonication for 1 h. Each solution loading of 100 μL/cm^2^ using a micropipette was cast onto CFP and allowed to dry in air overnight.

***Structural characterization:*** The morphology of the prepared materials was examined by field-emission scanning electron microscopy (FESEM) using a JEOL JSM-7500F instrument. Particles harvested from carbon fiber paper (CFP) substrate by ultrasonication in toluene and hydrothermally grown particles were examined by high-resolution transmission electron microscopy (HRTEM) using a JEOL JEM-2010 instrument operated at an accelerating voltage of 200 keV. Phase assignment was performed with the help of X-ray diffraction (XRD) using a Bruker D8-Advance instrument equipped with a Cu Kα source (λ = 1.5418 Å) as well as by Raman microprobe analysis using a Jobin-Yvon HORIBA LabRAM HR800 instrument coupled to an Olympus BX41 microscope. Raman spectra were collected with excitation from the 514.5 nm line of an Ar-ion laser; the laser power was kept below 10 mW to minimize photooxidation. Differential scanning calorimetry (DSC) analysis was performed using a TA Instruments Q2000 instrument. The temperature was scanned from 0 to 100 °C and back again to 0 °C at ramp rates ranging from 1 to 15 °C/min. For DSC experiments, the VO_2_-deposited CFP was cut into small pieces and stacked in an aluminum T-Zero pan under a purge flow of Ar gas. A bare CFP was used as a reference. The chemical composition and oxidation states of the prepared materials were investigated by X-ray photoelectron spectroscopy (XPS, Omicron XPS) with Mg Kα radiation (1253.6 eV). Energy calibration was achieved by setting the C1s line from adventitious hydrocarbons to 284.8 eV. Vanadium K-edge X-ray absorption near-edge structure (XANES) spectra were collected at the Advanced Light Source (ALS) bending magnet beamline 10.3.2. V K-edge XANES spectra were recorded in fluorescence mode in the energy range 5450–5600 eV by continuously scanning a Si (111) monochromator (Quick XAS mode) from 20 eV below to 40 eV above the white line absorption. For XANES analysis, a suite of custom LabVIEW programs at the beamline was used to perform deadtime correction, energy calibration, glitch removal, pre-edge subtraction, and post-edge normalization. The Athena suite of programs in the IFEFFIT package was used to analyze the XANES spectra.

***Electrochemical characterization:*** The oxygen evolution reaction (OER) performance of the prepared materials was evaluated using a three-electrode cell with the help of a Bio-Logic potentiostat (SP-200). All of the measurements were performed in a 1 M aqueous solution of KOH purged with N_2_ gas. The vanadium oxides prepared on CFP were individually used as the working electrodes. A saturated calomel electrode (SCE) and a Pt foil were used as reference and counter electrodes, respectively. The potential versus SCE (E_SCE_) was converted to the potential versus the reversible hydrogen electrode (RHE) (E_RHE_) using the relation E_RHE_ = E_SCE_ + 1.0464 V [41]. Polarization curves for OER were measured using linear sweep voltammetry (LSV) in the range between 1.2 and 1.8 V versus RHE at a scan rate of 8 mV/s. The polarization curves were corrected for the ohmic potential drop (i*R*) losses, where *R* is the series resistance of the electrochemical cell as determined by electrochemical impedance spectroscopy (EIS) measurements. EIS measurements were performed in the range between 200 kHz and 50 mHz using an AC amplitude of 25 mV. The double-layer capacitance (C_dl_) of the samples was determined by cyclic voltammetry (CV) at scan rates between 20–100 mV/s. Gas chromatography (GC) analysis of gaseous product was performed on the headspace of sealed electrocatalytic cells with the three-electrode configuration in a 1 M KOH electrolyte solution. The cells were sealed under an Ar ambient. After application of a constant voltage of 1.6 V versus RHE for 30 min, the headspace was sampled using a syringe. An Agilent Trace 1300 GC equipped with a thermal conductivity detector and a custom-made 120 cm stainless steel column packed with Carbosieve-II from Sigma-Aldrich was used for analysis. The carrier gas was Ar. Identification of O_2_ produced from electrolysis were accomplished by withdrawing 200 μL of the headspace using a 0.5 mL Valco Precision Sampling Syringe, Series A-2 equipped with a Valco Precision Sampling syringe needle with a five-point side port.

## 3. Results and Discussion

**Figure 1:**
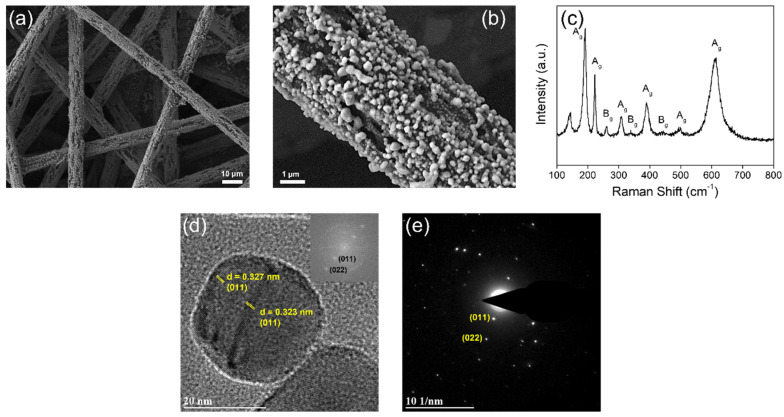
(**a**) a low-magnification and (**b**) high-magnification FESEM images and (**c**) room-temperature Raman spectrum acquired for the VO_2_ (M1) nanoparticles formed on carbon fiber paper (514.5 nm laser excitation). (**d**) Lattice-resolved HRTEM image and (**e**) the indexed SAED pattern of the VO_2_ (M1) nanoparticles harvested from the VO_2_/CFP sample by ultrasonication for 1 h in toluene. An individual VO_2_ (M1) nanoparticle shows an interplanar separation of 0.323–0.327 nm corresponding to the spacing between (200) lattice planes. The inset of (**d**) indicates its fast Fourier transform (FFT) image.

The VO_2_ nanoparticles have been prepared through the vacuum annealing of the V_2_O_5_ particles coated on a carbon fiber paper (CFP) at 950 °C, which is slightly higher than the melting point of V_2_O_5_ (690 °C at standard temperature and pressure). The field-emission scanning electron microscopy (FESEM) image in Figure 1a shows clearly that the produced VO_2_ nanoparticles with the size of *ca.* 300 ± 76 nm in diameter uniformly cover the surface of individual carbon fibers (*ca*. 7 μm in diameter) constituting the CFP. The bare surface of CFP is comparatively shown with a smooth grain by FESEM images in Figure S1 of the Supplementary material. More specifically, the VO_2_ nanoparticles have some particle coalescence, which results from solidifying after slightly melting down (see the high-magnification image in Figure 1b). The phase of VO_2_ nanoparticles obtained should be defined since the VO_2_ possesses three polymorphs, M1 monoclinic, M2 monoclinic, and R rutile phases, across the phase transition at *ca.* 67 °C in the bulk [34,35,36,37]. Each of them has distinctive Raman spectral signatures arising from the pronounced differences in local symmetry, where the space groups for the R and M1 phases are *P4_2_/mnm* (*D_4h_^14^*) and *P2_1_/c* (*C_2h_^3^*), respectively [36,37]. In particular, the most stable M1 phase at room temperature is characterized by 18 Raman-allowed modes, 9 each of A_g_ and B_g_ symmetry [36,37,42,43]. In Figure 1c, the Raman modes acquired for our VO_2_ nanoparticles at room temperature are assigned to the M1 monoclinic phase of VO_2_. The mode assignments denoted in Figure 1c are derived from group theory considerations and previously reported polarized Raman spectroscopy studies [42,43,44]. Furthermore, the XRD reflections acquired on CFP are indexed well to the M1 phase of VO_2_ (Joint Committee on Powder Diffraction Standards (JCPDS) 43-1051), as shown in Figure S2. These results indicate that the VO_2_ nanoparticles obtained here have a well-defined single M1 phase without any mixed phase.

The structural characterization has been further performed using high-resolution transmission electron microscopy (HRTEM) and selected area electron diffraction (SAED). Figure 1d,e show the lattice-resolved HRTEM image and the indexed SAED pattern of an individual VO_2_ (M1) nanoparticle harvested from the VO_2_/CFP sample by ultrasonication for 1 h in toluene (low-magnification TEM image of VO_2_ (M1) nanoparticles is shown in Figure S3a). The nanoparticle reveals the d-spacing of (011) lattice planes, which are the XRD main reflection of VO_2_ M1 monoclinic phase. The (200) lattice planes of VO_2_ M1 phase are also observed in another individual nanoparticle (Figure S3b). These results corroborate the single crystalline nature of the individual nanoparticles.

**Figure 2:**
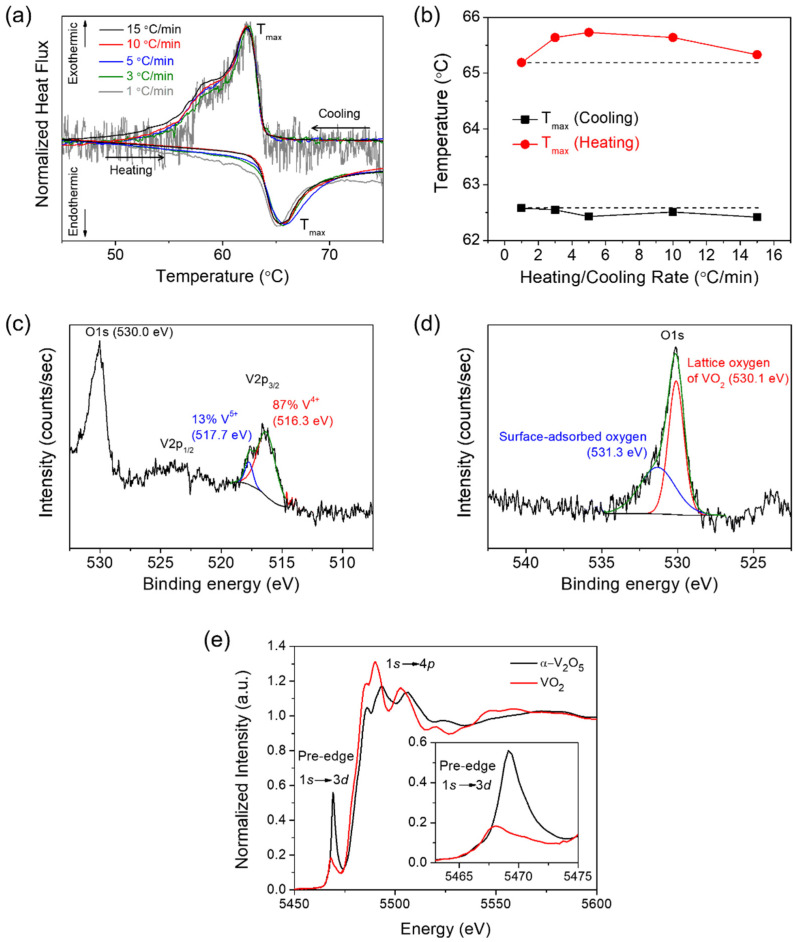
(**a**) Scan rate-dependent DSC results for the VO_2_ (M1) nanoparticles formed on carbon fiber paper. Various scan rates with 15, 10, 5, 3, and 1 °C/min were applied. (**b**) Evolution of T_max_ for the VO_2_ (M1) nanoparticles formed on carbon fiber paper, as a function of the heating/cooling rate. XPS spectra indicating (**c**) V 2p and (**d**) O 1s binding energies acquired for the VO_2_ (M1) nanoparticles formed on carbon fiber paper. The V 2p_3/2_ peak in (**c**) and O 1s peak in (**d**) are deconvoluted into respective two sub-peaks. (**e**) Experimental V K-edge XANES spectra acquired for the VO_2_ (M1) nanoparticles and α-V_2_O_5_ nanowires. The inset indicates the magnification of pre-edge peaks.

Meanwhile, it should be noticed that vanadium oxides have multiple vanadium oxidation states (V^2+^, V^3+^, V^4+^, V^5+^) [24,25]. Among them, VO_2_ with the oxidation state of V^4+^ exhibits the characteristic phase transition phenomenon at near 67 °C as mentioned earlier, which is distinct from compounds with the other oxidation states. Such property can be used for characterizing the phase and defect of the prepared VO_2_. In detail, the thermally and electrically induced metal-insulator phase transition in VO_2_ is accompanied by a considerable consumption (M1 → R) or release (R → M1) of latent heat, given that it is a first-order transition [37,45]. The latent heat at the phase transition comprises an enthalpy component arising from the structural distortion of the lattice (which is compensated in part by a modulation of the phonon entropy) and conduction entropy of electrons [37,45,46,47]. Figure 2a shows differential scanning calorimetry (DSC) profiles acquired at various scan rates for the prepared VO_2_ nanoparticles. The pronounced endothermic (M1 → R) and exothermic (R → M1) DSC traces centered at around 65 °C and 62 °C are observed upon heating and cooling, respectively; the temperatures are recognized at T_max_ which represents the temperature at the maximum height of the transition peak and is indicative of the maximum transformation rate [45]. Such DSC traces corroborate again that the prepared vanadium oxide is indeed VO_2_ with the metal-insulator phase transition. Figure 2b shows the evolution of hysteresis, which is defined as the difference between T_max_ temperatures acquired upon heating and cooling across the scan rates. The T_max_ upon heating is slightly altered between 65.2 °C and 65.7 °C across the measured scan rates, while the one upon cooling is almost constant with the values between 62.4 °C and 62.6 °C. As a result, it is found that the width of hysteresis in the prepared VO_2_ nanoparticles here is relatively scan-rate-invariant compared to that of the hydrothermally prepared VO_2_ nanoparticles previously [45]. The phase transitions in VO_2_ upon heating and cooling are known to be mediated by defects such as oxygen vacancy, twin boundary, and grain boundary, which serve as phase nucleation sites [45,48]. The extent of supercooling of the high-temperature phase and superheating of the low-temperature phase (thermal hysteresis) can be decreased with increasing concentration of defects since the nucleation probability is increased with increasing defect density. Therefore, the low thermal hysteresis for the prepared VO_2_ nanoparticles here indicates importantly that those nanoparticles possess a considerable number of structural defects.

The oxidation state and stoichiometry of VO_2_ have been characterized by X-ray photoelectron spectroscopy (XPS). Figure 2c,d show V 2p and O 1s spectra for the VO_2_ nanoparticles, respectively. The V 2p_3/2_ spectrum can be deconvoluted into two sub-peaks arising from the V^4+^ (516.3 eV) and V^5+^ (517.7 eV) components in Figure 2c, where the binding energy values for the spectra correspond to the reported values [49,50,51]. Noticeably, it indicates that the prepared VO_2_ consists primarily of V^4+^ component (87 at%) with the small amount of V^5+^ component (13 at%). The main peak for O 1s located at 530.1 eV is assigned to the lattice oxygen comprising the VO_2_ in Figure 2d [49,52]. The additional oxygen component located at 531.3 eV can be assigned to the surface-adsorbed oxygen or the C-O and C=O bonds coming from it [52]. Figure 2e plots V K-edge X-ray absorption spectra of the prepared VO_2_ and contrasting α-V_2_O_5_, acquired by X-ray absorption near-edge structure (XANES) spectroscopy. For comparison, α-V_2_O_5_ has been prepared in the form of nanowires by the hydrothermal method with the average diameters of between 150–250 nm and lengths ranging from 1–100 μm, according to a previously reported method [39]. The spectra consist of the pre-edge and white-line absorption features due to dipole-allowed transitions from V 1s to 3d states and from V 1s to 4p states, respectively. In Figure 2e, the VO_2_ (composed of V^4+^ ions) exhibits the characteristic V K-edge absorption spectrum with obviously different line shape, intensity, and peak position from α-V_2_O_5_ (composed of V^5+^ ions), indicating the discrepancy in the local symmetry and oxidation state of vanadium atoms between two types of V-O systems [53]. Specifically, a broader pre-edge peak for VO_2_ is observed at 5468.04 eV, shifting toward lower energy as compared to that of α-V_2_O_5_ (5469.13 eV) (see the inset of Figure 2e). On the other hand, a primary edge peak in the white-line absorption feature is more intensely observed at 5489.94 eV for VO_2_. A distinctive pre-edge feature observed in V K-edge spectrum of α-V_2_O_5_ is essentially local in character and originates from the broken octahedral symmetry of the vanadium centers in V_2_O_5_ [53]. The asymmetric broadening and shift of the area-weighted centroid to lower energy in the pre-edge feature across two V-O systems is a result of the reduction of the V^5+^ sites to V^4+^ sites. Upon such a reduction of vanadium sites, the electron remains localized within V 3d_xy_ orbitals with stabilization of a small polaron and the excitation of core electrons requires less energy due to more screening charge at the excited atom, and thus the pre-edge feature is shifted to lower energy [21,22,53]. In addition to the red shift of the pre-edge peak position, the V^4+^ character contributes to the broadening of the pre-edge peak due to the increase of octahedral symmetry precluding V 4p-3d hybridization, as shown in the inset of Figure 2e. As a result, it is found that the prepared VO_2_ is considerably stoichiometric and somewhat free from the point defect such as oxygen vacancy. Hence, the principal defect associated with the low thermal hysteresis across the phase transition for VO_2_ nanoparticles is considered grain boundaries among VO_2_ nanoparticles with some particle coalescence, as observed in FESEM image of Figure 1a.

To investigate the effect of vanadium oxidation state (V^4+^/V^5+^) on the electrocatalytic properties of vanadium oxide, we have further prepared V_2_O_5_ nanoparticles covering the carbon fibers through the annealing of the V_2_O_5_ particles coated on the CFP at 950 °C under an O_2_ flow of 10 sccm and continuous vacuum pumping condition using a tube furnace. FESEM images in Figure S4a,b reveal the morphology of the V_2_O_5_ nanoparticles covering uniformly the surface of individual carbon fibers constituting the CFP, where the morphology and particle size are similar to those of the VO_2_ nanoparticles. Only slightly faceted shape in the V_2_O_5_ nanoparticles is observed as compared with the VO_2_ nanoparticles (Figure S4b). The Raman bands acquired for the prepared V_2_O_5_ nanoparticles in Figure S4c are well matched with the Raman-active modes of V_2_O_5_ reported in the literature [54,55]. XPS V 2p and O 1s spectra for the prepared V_2_O_5_ nanoparticles are shown in Figure S4a,b, respectively. The XPS result demonstrates that the prepared V_2_O_5_ nanoparticles are composed primarily of V^5+^ component (80 at%) with the small amount of V^4+^ component (20 at%). Besides, VO_2_ (M1), α-V_2_O_5_, and γ′-V_2_O_5_ have been separately prepared by the hydrothermal methods for comparison, followed by drop-casting onto CFP. The agglomeration of the hydrothermally grown VO_2_ (M1) nanoparticles in a few nanometer size is shown by TEM in Figure S5a,b. The hydrothermally grown VO_2_ (M1) nanoparticles are characterized to possess the same ratio of V^4+^ to V^5+^ by XPS (Figure S5c,d), which is a higher amount of V^5+^ component compared to the VO_2_ nanoparticles prepared by the vacuum annealing. The α-V_2_O_5_ nanowires with the average diameters of between 150–250 nm and lengths ranging from 1–100 μm and the metastable γ′-V_2_O_5_ polymorph microrods with average widths of 0.9–1.1 μm were synthesized separately (their XRD patterns and FESEM images are shown in Figure S6).

**Figure 3:**
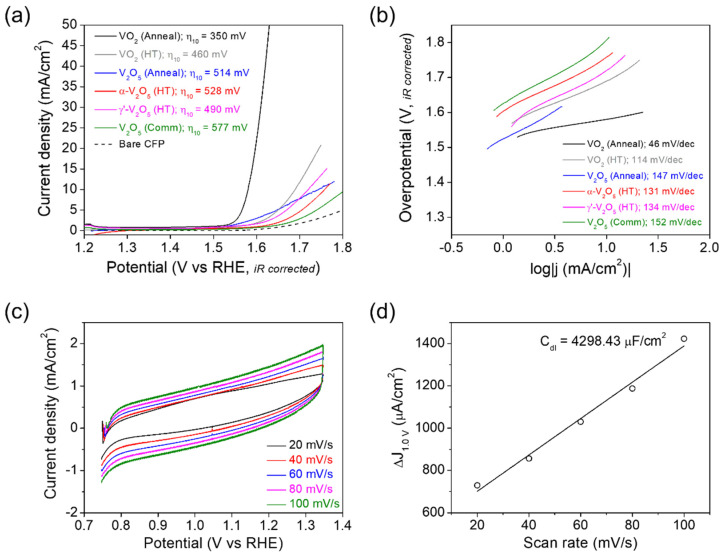
(**a**) Polarization curves and (**b**) Tafel plots measured for the vacuum-annealed VO_2_ (M1) and V_2_O_5_, hydrothermally grown VO_2_ (M1), α-V_2_O_5_, and γ′-V_2_O_5_, and commercial V_2_O_5_, prepared on carbon fiber paper, contrasted to data acquired for bare CFP. The data have been acquired in aqueous solutions of 1 M KOH using a three-electrode assembly. (**c**) Cyclic voltammograms acquired at various scan rates for the VO_2_ (M1) nanoparticles formed on carbon fiber paper. (**d**) The differences in current density (*Δj = j_a_ − j_c_*) at 1.0 V versus RHE are plotted as a function of the scan rate. The C_dl_ value is extrapolated from a linear fit to the plot.

The electrocatalytic OER characteristics of the vanadium oxides prepared on CFP have been investigated in a 1 M aqueous solution of KOH, using a conventional three-electrode setup. Figure 3a displays linear polarization curves, which have been corrected for ohmic potential drop (iR) losses. Bare CFP is contrasted as a control and is essentially catalytically inert toward OER. Remarkably high OER performance for the VO_2_ (M1) nanoparticles prepared by the vacuum annealing on CFP is found with an overpotential of 350 mV, reaching a current density of 10 mA/cm^2^ (η_10_) and a Tafel slope of 46 mV/dec (Figure 3b). The hydrothermally grown VO_2_ (M1) nanoparticles prepared by drop-casting on CFP exhibit the second-highest OER activity with a η_10_ value of 460 mV and a Tafel slope of 114 mV/dec. As compared to the VO_2_ samples, V_2_O_5_ samples represent relatively low OER activities with higher η_10_ values more than 490 mV and higher Tafel slope values more than 130 mV/dec. Among the V_2_O_5_ samples, γ′-V_2_O_5_ shows the lowest η_10_ value of 490 mV, but its Tafel slope value (134 mV/dec) is roughly equal to that of α-V_2_O_5_ (131 mV/dec), indicating their same OER kinetics.

We further measured the double layer capacitance (C_dl_) for the samples using a cyclic voltammetry (CV) method to estimate the electrochemically active surface area (ECSA), which is directly proportional to C_dl_ [18,33]. The voltammograms for the VO_2_ (M1) nanoparticles prepared by the vacuum annealing on CFP have been collected at various scan rates (20–100 mV/s) in the potential range of 0.75–1.35 V versus reversible hydrogen electrode (RHE) (a potential range with no Faradaic current), where the current is preponderantly due to the charging of the double layer (and not due to water oxidation), as shown in Figure 3c. The differences (*Δj*) of anodic and cathodic current densities at 1.0 V vs. RHE for the CV plot are shown as a function of the scan rate in Figure 3d. The slope of *Δj* vs. scan rate plot is equal to a value of 2C_dl_. The CV and *Δj* vs. scan rate plots acquired for the α-V_2_O_5_, γ′-V_2_O_5_, and commercial V_2_O_5_ samples prepared on CFP are shown in Figure S7 for comparison. As a result, the VO_2_ (M1) nanoparticles prepared by the vacuum annealing, which have the lowest η_10_ (350 mV) and Tafel slope (46 mV/dec) values, are found to exhibit the highest C_dl_ value of 4298.43 μF/cm^2^. By contrast, α-V_2_O_5_, γ′-V_2_O_5_, and commercial V_2_O_5_ show relatively low C_dl_ values of 742.54, 748.98, and 491.42 μF/cm^2^, respectively (Figure S7). These experimental results definitely indicate that VO_2_ shows higher OER activity than V_2_O_5_. In other words, it is concluded that V^4+^ in vanadium oxide composes a catalytically active site for the OER with high intrinsic activity. A much higher OER activity of the VO_2_ (M1) nanoparticles prepared directly on CFP by the vacuum annealing compared to the hydrothermally grown and drop-casted ones can be explained by their improved coverage and adhesion on the carbon fibers (i.e., improved morphological factor by process innovation, facilitating charge transfer) as well as their higher V^4+^ content (87% for the former and 50% for the latter, according to the XPS result). For V_2_O_5_ catalysts, a slightly higher OER activity (lower η_10_) of γ′-V_2_O_5_ than α-V_2_O_5_, despite their identical OER mechanism with the roughly same Tafel slope value, is regarded to result from a smaller particle size and concurrently higher ECSA (higher C_dl_ value) of γ′-V_2_O_5_ than α-V_2_O_5_ (Figure S6). Indeed, much bulkier commercial V_2_O_5_ particles with the lowest ECSA (lowest C_dl_) show the lowest OER activity. However, it should be noted that these V^5+^-constituted V_2_O_5_ catalysts reveal much lower OER activities compared to the V^4+^-constituted VO_2_ catalyst. According to the theoretically proposed mechanism, the OER proceeds in the four steps in a basic environment as per [8]:

4OH^−^ → OH * + 3OH^−^ + e^−^ (1)

OH * + 3OH^−^ → O * + 2OH^−^ + H_2_O + e^−^ (2)

O * + 2OH^−^ + H_2_O → OOH * + OH^−^ + H_2_O + e^−^ (3)

OOH * + OH^−^ + H_2_O → O_2_ + 2H_2_O + e^−^ (4)

where * denotes a surface adsorption site. The adsorption energies for intermediates of OH *, O *, and OOH *, formed in the reaction steps, determine the efficiency of the catalyst; i.e., the lowest overpotential is achieved when the energies of the OH * → O * (Equation (2)) and O * → OOH * (Equation (3)) steps are equalized. Thus, the lower energy disparity between two steps on the adsorption sites of the catalyst is, the higher efficiency of the catalyst is. Meanwhile, the entire water-splitting cycle can be divided into a metal oxidation step and metal reduction step with O_2_ evolution, where the oxidation state and redox kinetics of the transition metal play important roles in determining the OER efficiency by affecting the adsorption energy [8]. When the kinetics of the metal reduction with O_2_ evolution step is slow or rapid, the metal oxidation maintains a high-valent or low-valent state at the OER potential, respectively. Here, when the kinetics of the metal-reduction step is controlled to maintain the metal oxidation state with a high intrinsic OER activity at the OER potential, the OER activity of the catalyst is enhanced. Therefore, in this work, the high OER activity of VO_2_ is attributed to the preservation of highly OER active V^4+^ components and V^4+/5+^ redox couples in VO_2_. Likewise, V^3+^, Co^3+^, Fe^3+^, Ni^3+^, and their redox couples are known to work as the OER-active species in certain crystal structures [8,18]. More recently, the amorphous VO_x_ and CoVO_x_ with V^4+^ were also reported to have high OER activities [9]. A high OER activity of the VO_2_ (M1) with η_10_ of 350 mV and Tafel slope of 46 mV/dec is found to be comparable to those of various electrocatalysts reported in the literature (see Table S1): e.g., η_10_ of 390 mV for CoO_x_, η_10_ of 465 mV for amorphous VO_x_, η_10_ of 410 mV for V_2_O_5_, and 534 mV for Co_3_O_4_. In particular, the VO_2_ (M1) shows a much lower level of Tafel slope value compared to the nickel-, cobalt-, and manganese-based catalysts as well as the other vanadium-based catalysts such as VOOH, amorphous VO_x_, and V_6_O_13_. The η_10_ of VO_2_ (M1) is even comparable to those of state-of-the-art OER catalysts, RuO_2_ and IrO_2_ with η_10_ of around 300 mV.

**Figure 4:**
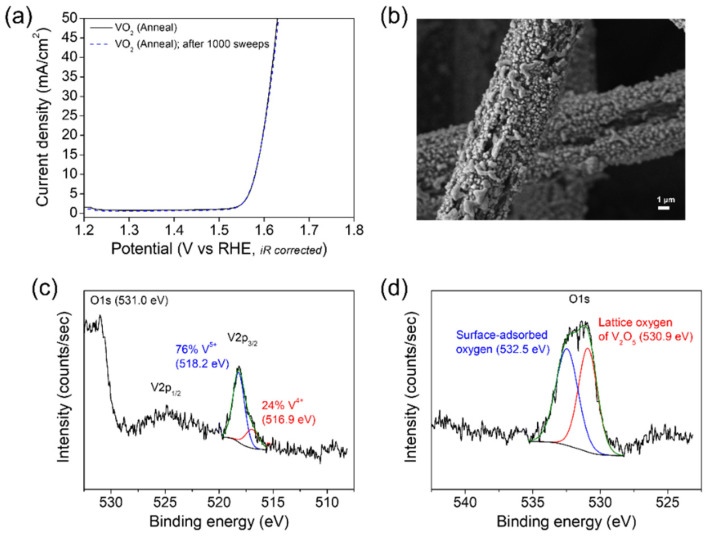
(**a**) Polarization curves of the VO_2_ (M1) nanoparticles prepared by the vacuum annealing on CFP recorded before and after 1000 CV sweeps in a 1 M aqueous solution of KOH. (**b**) FESEM image acquired for the VO_2_ (M1) nanoparticles after 1000 CV sweeps. XPS spectra of (**c**) V 2p and (**d**) O 1s acquired for the surface of the VO_2_ (M1) catalyst after OER under a constant voltage of 1.6 V versus RHE for 30 min in a 1 M KOH electrolyte solution on the headspace of the electrocatalytic cell sealed under an Ar ambient with a three-electrode system.

The oxygen evolution from the VO_2_ (M1) nanoparticles prepared by the vacuum annealing on CFP has been corroborated by gas chromatography (GC) analysis. The resulting GC trance in Figure S8 shows the only peak eluting from the column at 2.29 min, corresponding to O_2_. The long-term stability test of the VO_2_ (M1) catalyst has been performed by 1000 repeated CV sweeps in a 1 M aqueous solution of KOH in the range between 0.75 and 1.60 V versus RHE at a scan rate of 100 mV/s. As sown in Figure 4a, the VO_2_ (M1) catalyst exhibits stable performance with almost exactly superimposable polarization curves after 1000 sweeps. The FESEM image of the catalyst acquired after the long-term stability test is shown in Figure 4b. Compared with the morphology before 1000 sweeps in Figure 1b, there seemed to be no remarkable difference. To further investigate the surface state of the VO_2_ (M1) catalyst after OER, XPS spectra were acquired for the catalyst after OER under a constant voltage of 1.6 V versus RHE for 30 min in a 1 M KOH electrolyte solution (Figure 4c,d). Noticeably, the V^5+^ component on the surface of the catalyst is found to be largely increased from 13% to 76% after OER, which is close to V_2_O_5_. Such an oxidation of vanadium during OER again corroborates a relatively poor OER activity of V^5+^ site in VO_2_ (M1), although the overpotential and morphology for the catalyst did not change obviously until 1000 CV sweeps. Similarly, the metal oxidation behavior involving a performance degradation is found in the several literature reports. For example, the surface of VOOH catalyst is observed to oxidize from V^3+^ to V^5+^ after OER [18]. Also, Kim et al. reported the surface oxidation from Co^2+^ to Co^3+^ after OER in amorphous cobalt phyllosilicate catalyst [14].

The phase transition of VO_2_ was investigated in this work only to identify an accurate preparation of the VO_2_ (M1) nanoparticles. Since the phase transition temperature was found to be *ca.* 65 °C (heating) and 62 °C (cooling), the effect of phase transition on the OER in electrocatalytic systems operating at room temperature was ignored. Although the relationship between phase transition and electrocatalytic (including the OER) activity in VO_2_ goes beyond the subject of this study, it can be another huge topic requiring further investigation.

## 4. Conclusions

In summary, in this work, the VO_2_ (M1) is found to possess high electrocatalytic OER activity and stability for the first time. For this, the single-phase VO_2_ (M1) nanoparticles, uniformly covering the surface of individual carbon fibers constituting the CFP, have been prepared through the vacuum annealing technique. The thermal analysis corroborates that the prepared VO_2_ nanoparticles reveal the metal-insulator phase transition at *ca.* 65 °C (heating) and 62 °C (cooling) with the low thermal hysteresis, indicating a high concentration of structural defect which is considered grain boundaries among VO_2_ nanoparticles with some particle coalescence. Consequently, the prepared VO_2_ (M1) nanoparticles on CFP show a high electrocatalytic OER activity with the lowest η_10_ (350 mV) and Tafel slope (46 mV/dec) values in a 1 M aqueous solution of KOH as compared to those of the vacuum annealed V_2_O_5_ and the hydrothermally grown VO_2_ (M1), α-V_2_O_5_, and γ′-V_2_O_5_. The catalytically active site is considered V^4+^ components and V^4+/5+^ redox couples in VO_2_. The oxidation state of V^4+^ is revealed to be more favorable to the OER catalysis compared to that of V^5+^ in vanadium oxide through comparative studies. Furthermore, the amount of V^5+^ component is found to be increased on the surface of VO_2_ catalyst during OER, giving rise to the performance degradation. This work suggests V^4+^ and its redox couple as a novel active component for OER in metal-oxide electrocatalysts.

## Data Availability

Not applicable.

## Supplementary Materials

The following supporting information can be downloaded at: https://www.mdpi.com/article/10.3390/nano12060939/s1, References [9,10,14,18,19,20,56,57,58,59] are cited in the Supplementary Materials. Figure S1. FESEM images of a bare carbon fiber paper (CFP). (b) illustrates the high-magnification image of a single carbon fiber. Figure S2. XRD pattern of VO_2_ nanoparticles prepared on CFP. The reflections are indexed to VO_2_ M1 phase. The reflections denoted by CFP arise from the graphitized CFP substrate. The vertical bars indicate the reflections of VO_2_ M1 phase (Joint Committee on Powder Diffraction Standards (JCPDS) card# 43-1051). Figure S3. (a) Low-magnification TEM image of the VO_2_ (M1) nanoparticles harvested from the VO_2_/CFP sample by ultrasonication for 1 h in toluene. (b) Lattice-resolved HRTEM image of an individual VO_2_ (M1) nanoparticle showing an interplanar separation of 0.242 nm corresponding to the spacing between (200) lattice planes. The inset indicates its fast Fourier transform (FFT) image. Figure S4. (a) Low- and (b) high-magnification FESEM images, (c) Raman spectrum (514.5 nm laser excitation), and XPS (d) V 2p and (e) O 1s spectra of the V_2_O_5_ nanoparticles prepared on CFP. Figure S5. (a) Low-magnification TEM and (b) HRTEM images of the VO_2_ (M1) nanoparticles prepared by the hydrothermal method. XPS (c) V 2p and (d) O 1s spectra of the VO_2_ (M1) nanoparticles. Figure S6. Indexed XRD patterns for (a) α- and (b) γ’-V_2_O_5_ polymorphs. FESEM images for (c) α- and (d) γ’-V_2_O_5_. The α- and γ’-V_2_O_5_ have been prepared by the hydrothermal methods. Figure S7. Cyclic voltammograms acquired at various scan rates for (a) α-V_2_O_5_, (c) γ’-V_2_O_5_, and (e) commercial V_2_O_5_ prepared on CFP. The differences in current density (*Δj* = *j_a_* − *j_c_*) at 0.9 or 1.0 V *versus* RHE are plotted as a function of the scan rate for (b) α-V_2_O_5_, (d) γ’-V_2_O_5_, and (f) commercial V_2_O_5_ prepared on CFP. The C_dl_ values are extrapolated from a linear fit to the plot. Figure S8. Gas chromatogram (GC) of the generated O_2_ gas for the VO_2_ (M1) nanoparticles prepared by the vacuum annealing on CFP. For GC analysis, the O_2_ gas was captured after application of a constant voltage of 1.6 V *versus* RHE for 30 min in a 1 M KOH electrolyte solution on the headspace of the electrocatalytic cell sealed under an Ar ambient with a three-electrode system. Table S1. Comparison of the electrocatalytic OER activity for metal oxide catalysts.
