# Supplementary material for: VO2 as a Highly Efficient Electrocatalyst for the Oxygen Evolution Reaction
Source: Nanomaterials (Basel). 2022 Mar 12;12(6):939. doi: 10.3390/nano12060939 (PMC8951100; doi:10.3390/nano12060939)
Supplement: Supplementary file 1 [file nanomaterials-12-00939-s001.zip › nanomaterials-1609734-supplementary.pdf]

nanomaterials

Supplementary material

VO_2 as a Highly Efficient Electrocatalyst for the Oxygen Evolution Reaction

Yun-Hyuk Choi

School of Advanced Materials and Chemical Engineering, Daegu Catholic University, Gyeongsan 38430, Gyeongbuk, Korea; yunhyukchoi@cu.ac.kr

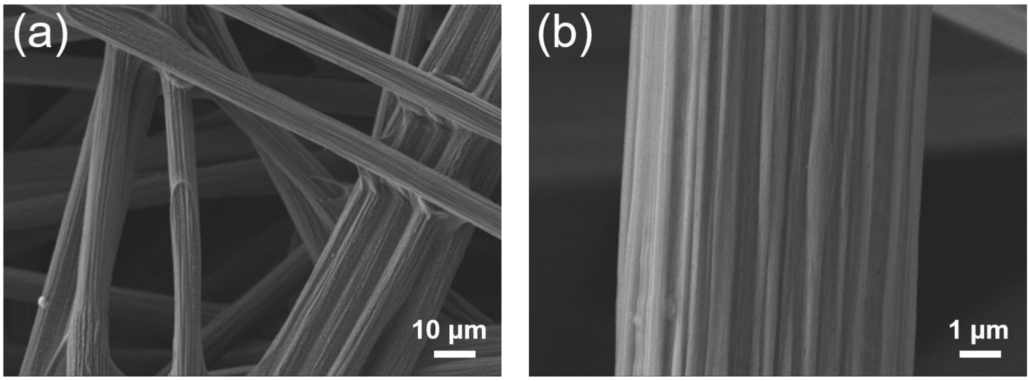

Figure S1. FESEM images of a bare carbon fiber paper (CFP). (b) illustrates the high-magnification image of a single carbon fiber.

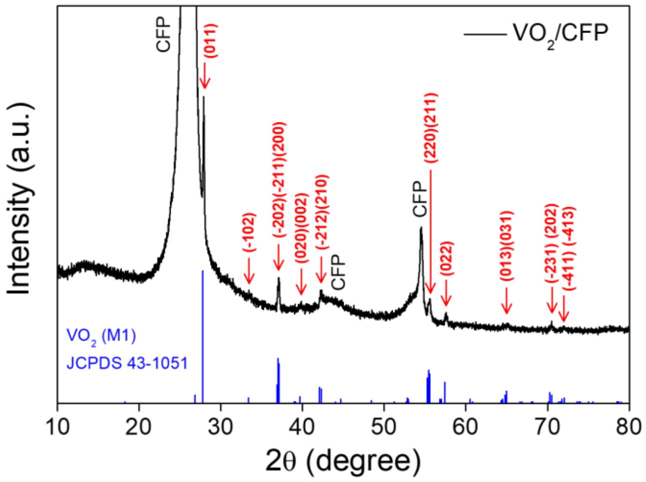

Figure S2. XRD pattern of VO_2 nanoparticles prepared on CFP. The reflections are indexed to VO_2 M1 phase. The reflections denoted by CFP arise from the graphitized CFP substrate. The vertical bars indicate the reflections of VO_2 M1 phase (Joint Committee on Powder Diffraction Standards (JCPDS) card# 43-1051).

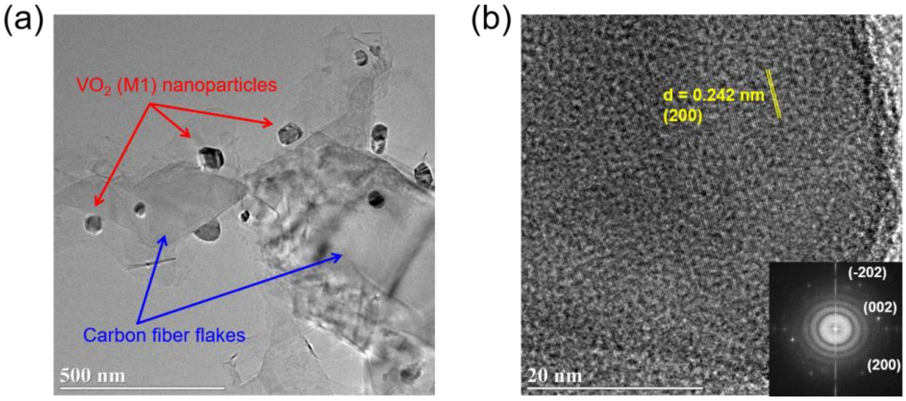

Figure S3. (a) Low-magnification TEM image of the VO_2 (M1) nanoparticles harvested from the VO_2/CFP sample by ultrasonication for 1 h in toluene. (b) Lattice-resolved HRTEM image of an individual VO_2 (M1) nanoparticle showing an interplanar separation of 0.242 nm corresponding to the spacing between (200) lattice planes. The inset indicates its fast Fourier transform (FFT) image.

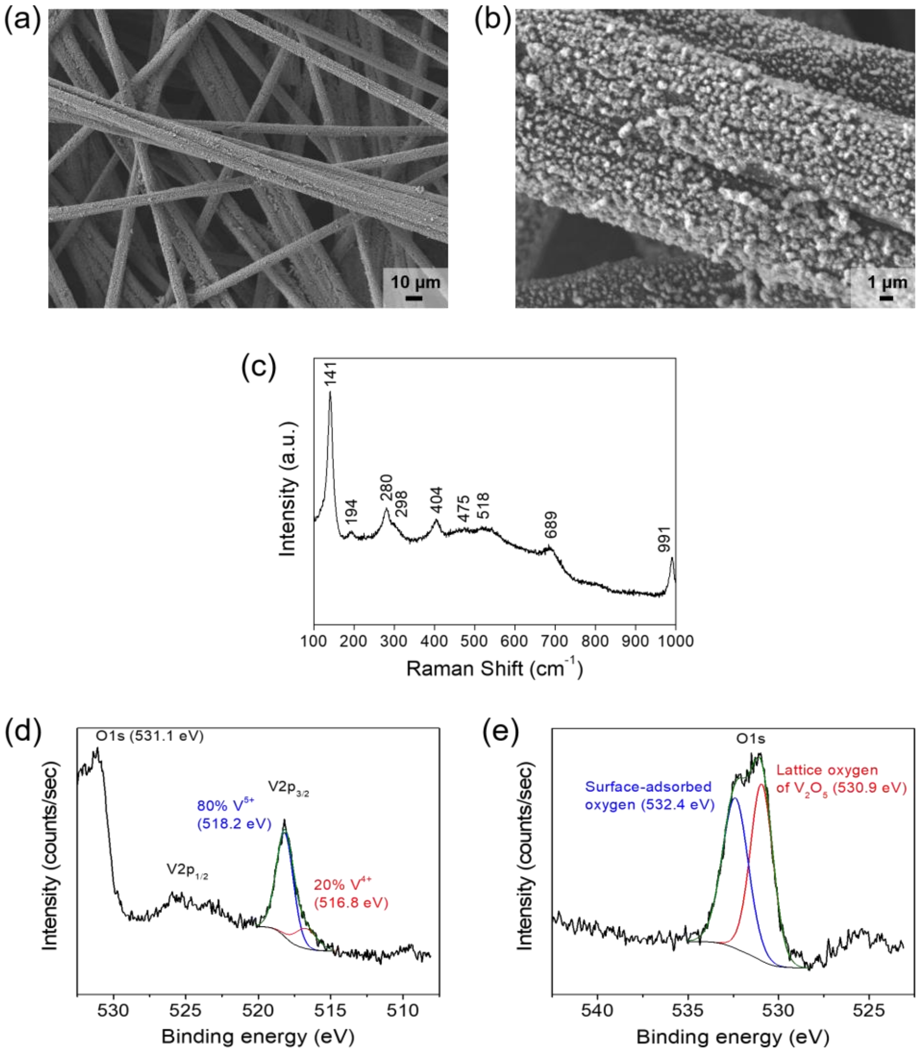

Figure S4. (a) Low- and (b) high-magnification FESEM images, (c) Raman spectrum (514.5 nm laser excitation), and XPS (d) V 2p and (e) O 1s spectra of the V_2O_5 nanoparticles prepared on CFP.

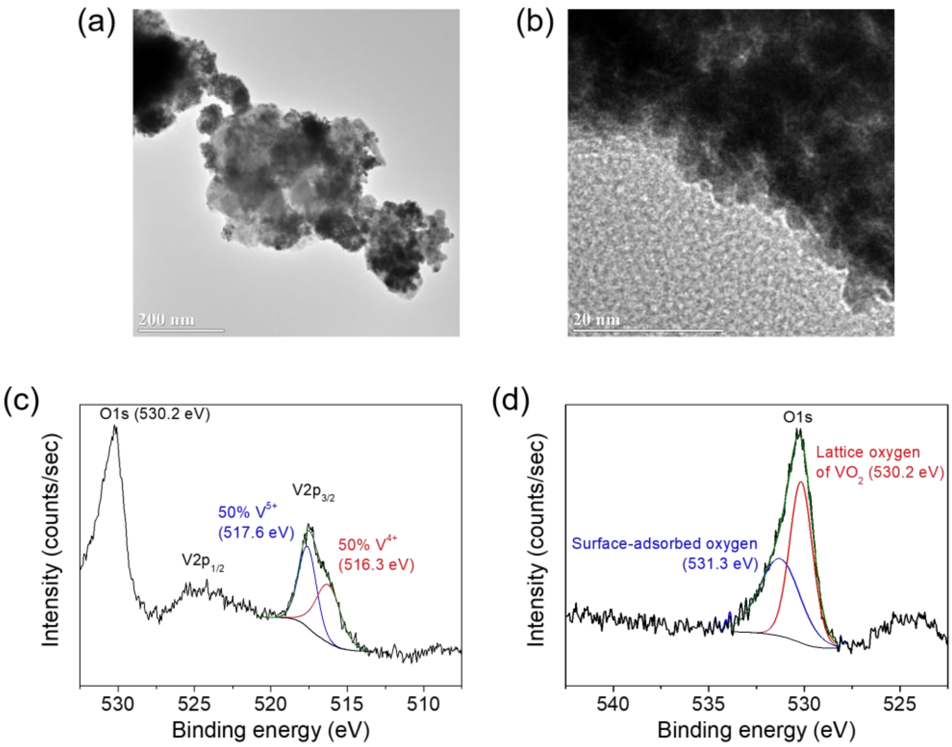

Figure S5. (a) Low-magnification TEM and (b) HRTEM images of the VO_2 (M1) nanoparticles prepared by the hydrothermal method. XPS (c) V 2p and (d) O 1s spectra of the VO_2 (M1) nanoparticles.

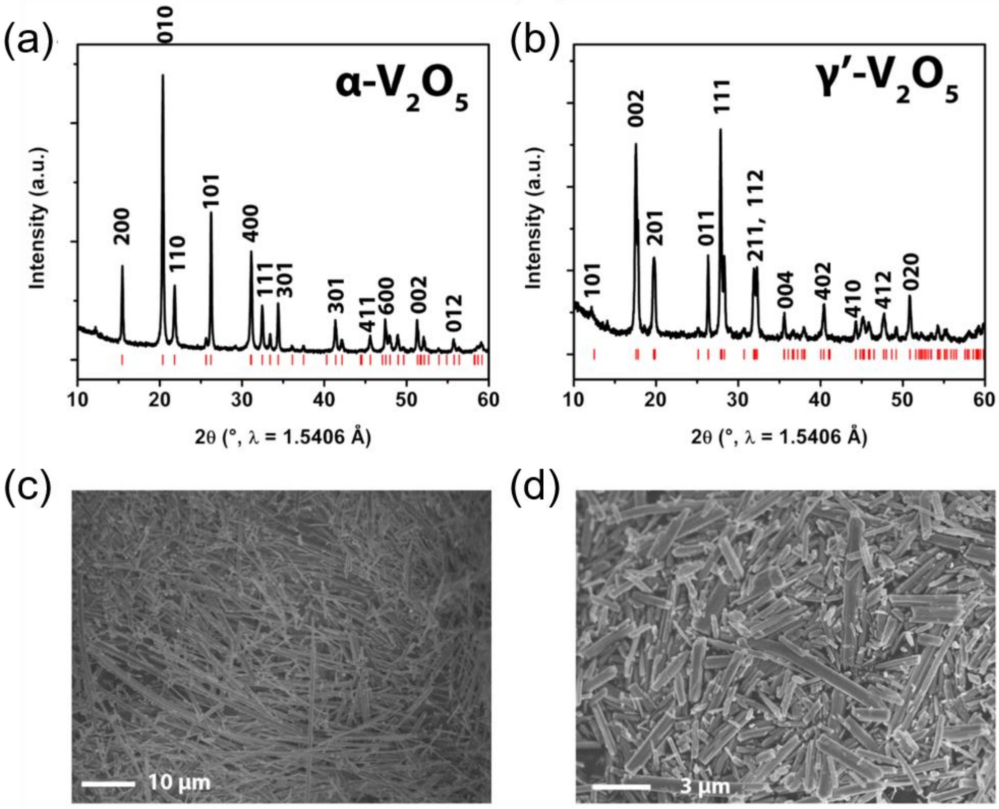

Figure S6. Indexed XRD patterns for (a) α- and (b) γ'-V_2O_5 polymorphs. FESEM images for (c) α- and (d) γ'-V_2O_5. The α- and γ'-V_2O_5 have been prepared by the hydrothermal methods.

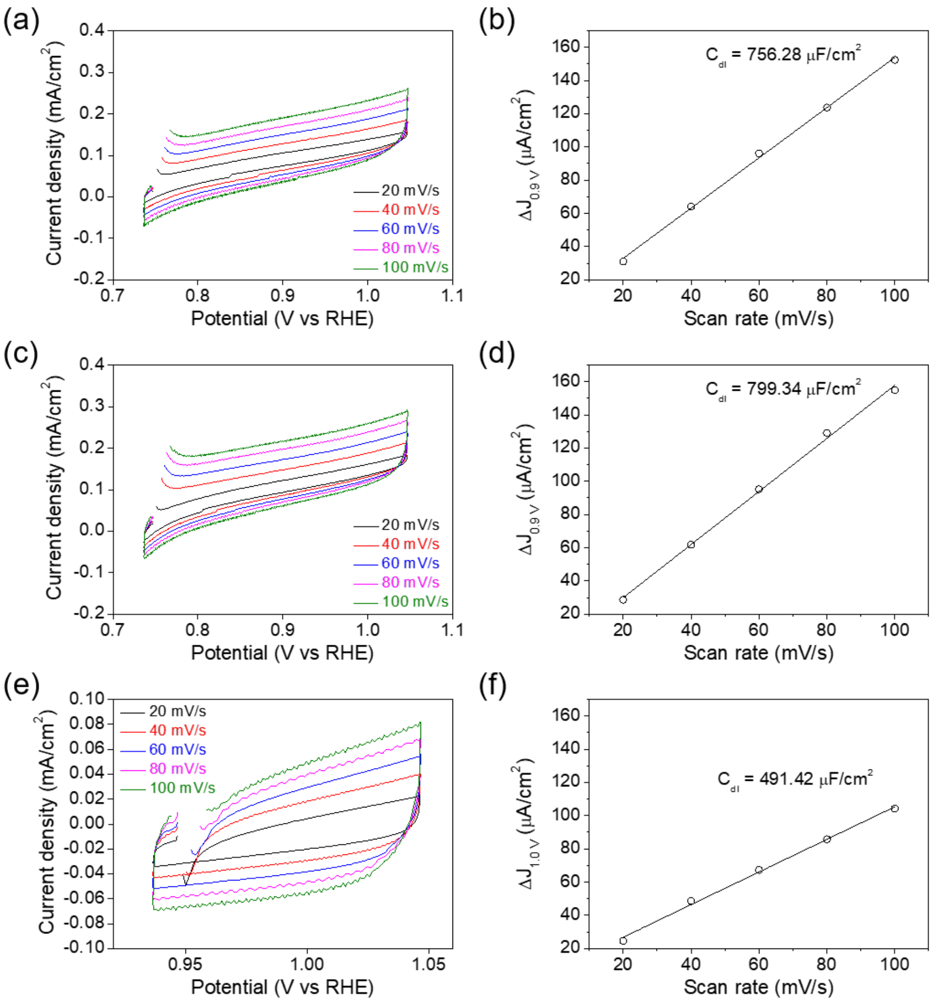

Figure S7. Cyclic voltammograms acquired at various scan rates for (a) α-V_2O_5, (c) γ′-V_2O_5, and (e) commercial V_2O_5 prepared on CFP. The differences in current density ($\Delta j = j_a - j_c$) at 0.9 or 1.0 V *versus* RHE are plotted as a function of the scan rate for (b) α-V_2O_5, (d) γ′-V_2O_5, and (f) commercial V_2O_5 prepared on CFP. The C_{dl} values are extrapolated from a linear fit to the plot.

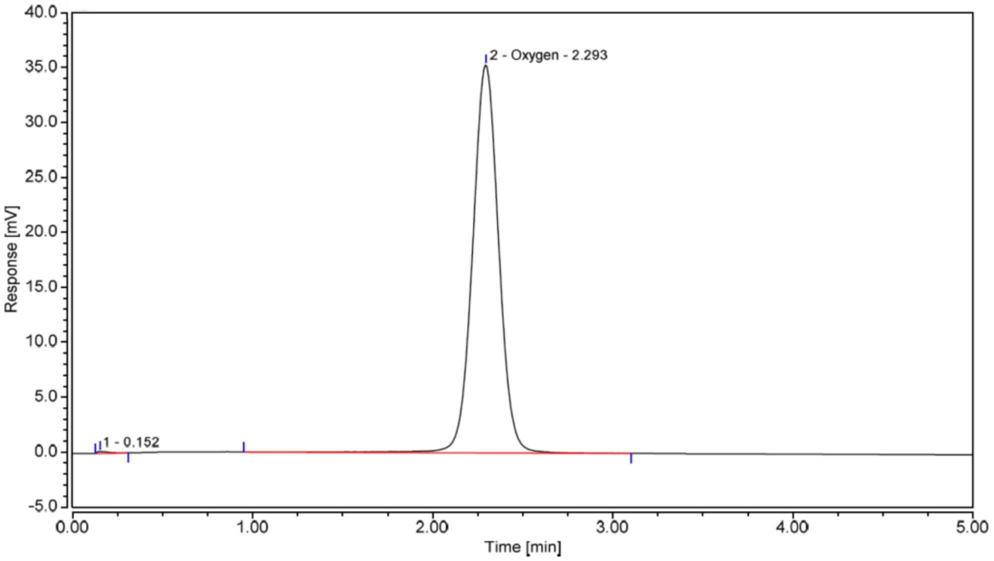

Figure S8. Gas chromatogram (GC) of the generated O_2 gas for the VO_2 (M1) nanoparticles prepared by the vacuum annealing on CFP. For GC analysis, the O_2 gas was captured after application of a

constant voltage of 1.6 V *versus* RHE for 30 min in a 1 M KOH electrolyte solution on the headspace of the electrocatalytic cell sealed under an Ar ambient with a three-electrode system.

Table S1. Comparison of the electrocatalytic OER activity for metal oxide catalysts.

Materials	Electrolyte (aqueous solution)	Overpotential (mV) reaching 10 mA/cm^2	Tafel slope (mV/dec)	Reference
IrO_x	1 M NaOH	320	N/A	[S1]
$NiFeO_x$	1 M NaOH	350	N/A	[S1]
$CoFeO_x$	1 M NaOH	370	N/A	[S1]
$NiCoO_x$	1 M NaOH	380	N/A	[S1]
CoO_x	1 M NaOH	390	N/A	[S1]
$NiLaO_x$	1 M NaOH	410	N/A	[S1]
$NiCuO_x$	1 M NaOH	410	N/A	[S1]
CoO_x/CoPi	1 M NaOH	420	N/A	[S1]
NiO_x	1 M NaOH	420	N/A	[S1]
$NiCeO_x$	1 M NaOH	430	N/A	[S1]
VOOH/Ni foam	1 M KOH	270	68	[S2]
VOOH/carbon paper	1 M KOH	321	82	[S2]
NiV LDH	1 M KOH	318	50	[S2]
NiFe LDH/CNT	1 M KOH	>300	31	[S2]
NiFe LDH	1 M KOH	302	40	[S2]
CoMn LDH	1 M KOH	324	43	[S2]
α-MnO_2	1 M KOH	490	77.5	[S2]
Co_3O_4/C	1 M KOH	290	70	[S2]
$NiCo_2O_4$	1 M KOH	290	53	[S2]
Amorphous cobalt phyllosilicate	1 M KOH	364	60	[S3]
CoOOH	1 M KOH	405	72	[S3]
Co_3O_4	1 M KOH	489	74	[S3]
NiCo LDH/carbon paper	1 M KOH	367	40	[S4]
Amorphous CoO_x/glassy carbon	1 M KOH	390	57	[S5]
Amorphous VO_x/glassy carbon	1 M KOH	465	97	[S5]
Amorphous $CoVO_x$ (Co:V = 3:1) /glassy carbon	1 M KOH	347	51	[S5]
Amorphous $CoVO_x$ (Co:V = 3:1) / Ni foam	1 M KOH	254	35	[S5]
Mn_3N_2/SnO_2:F	1 M KOH	390	97	[S6]
Mn_2O_3/SnO_2:F	1 M KOH	470	108	[S6]
Mn_3N_2/Ni foam	1 M KOH	270	101	[S6]
Mn_2O_3/Ni foam	1 M KOH	330	108	[S6]
RuO_2/Ni foam	1 M KOH	300	N/A	[S6]
IrO_2/Ni foam	1 M KOH	280	N/A	[S6]
IrO_2/SnO_2:F	1 M KOH	400	N/A	[S6]
NiFe LDH/glassy carbon	1 M KOH	280	49.4	[S7]

Amorphous CoV hydr(oxy)oxide /glassy carbon	1 M KOH	250	44	[S8]
Cobalt hydroxide /glassy carbon	1 M KOH	300	60	[S8]
Bulk crystalline CoV hydr(oxy)oxide /glassy carbon	1 M KOH	360	63	[S8]
V_2O_5/glassy carbon	1 M KOH	410	44	[S8]
IrO_2/glassy carbon	1 M KOH	330	48	[S8]
$Co_3V_2O_8$/glassy carbon	1 M KOH	359	65	[S9]
Co_3O_4/glassy carbon	1 M KOH	384	72	[S9]
V_2O_5/glassy carbon	1 M KOH	451	85	[S9]
Co-nanoparticles-embedded VN nanosheets /glassy carbon	1 M KOH	320	55	[S10]
$Co_2V_2O_7$/glassy carbon	1 M KOH	340	62	[S10]
IrO_2/glassy carbon	1 M KOH	370	62	[S10]
Co_3O_4	1 M KOH	534	47 ± 7	[S10]
Fe-doped $Co_3V_2O_8$ (V:Co:Fe = 3:4:3) /glassy carbon	1 M KOH	307	36	[S11]
$Co_3V_2O_8$/glassy carbon	1 M KOH	N/A	62	[S11]
$Fe_{2.5}V_{1.5}V_{5.6}O_{16}$ /glassy carbon	1 M KOH	N/A	54	[S11]
V_6O_{13}/glassy carbon	1 M KOH	N/A	101	[S11]
Fe_2O_3/glassy carbon	1 M KOH	N/A	209	[S11]
Co_3O_4/glassy carbon	1 M KOH	N/A	52	[S11]
VO_2 (anneal)/CFP	1 M KOH	350	46	This work
VO_2 (hydrothermal)/CFP	1 M KOH	460	114	This work
V_2O_5 (anneal)/CFP	1 M KOH	514	147	This work
α-V_2O_5 (hydrothermal) /CFP	1 M KOH	528	131	This work
γ'-V_2O_5 (hydrothermal) /CFP	1 M KOH	490	134	This work

References

S1. C. C. L. McCrory, S. Jung, J.C. Peters, T.F. Jaramillo, Bechmarking Heterogeneous Electrocatalysts for the Oxygen Evolution Reaction, *J. Am. Chem. Soc.* **2013**, *135*, 16977–16987.

S2. H. Shi, H. Liang, F. Ming, Z. Wang, Efficient Overall Water-Splitting Electrocatalysis Using Lepidocrocite VOOH Hollow Nanospheres, *Angew. Chem. Int. Ed.* **2017**, *56*, 573–577.

S3. J. S. Kim, I. Park, E.-S. Jeong, K. Jin, W.M. Seong, G. Yoon, H. Kim, B. Kim, K.T. Nam, K. Kang, Amorphous Cobalt Phyllosilicate with Layered Crystalline Motifs as Water Oxidation Catalyst, *Adv. Mater.* **2017**, *29*, 1606893.

S4. H. Liang, F. Meng, M. Cabán-Acevedo, L. Li, A. Forticaux, L. Xiu, Z. Wang, S. Jin, Hydrothermal Continuous Flow Synthesis and Exfoliation of NiCo Layered Double Hydroxide Nanosheets for Enhanced Oxygen Evolution Catalysis, *Nano Lett.* **2015**, *15*, 1421–1427.

S5. L. Liardet, X. Hu, Amorphous Cobalt Vanadium Oxide as a Highly Active Electrocatalyst for Oxygen Evolution, *ACS Catal.* **2018**, *8*, 644–650.

S6. C. Walter, P.W. Menezes, S. Orthmann, J. Schuch, P. Cannor, B. Kaiser, M. Lerch, M. Driess, A Molecular Approach to Manganese Nitride Acting as a High Performance Electrocatalyst in the Oxygen Evolution Reaction, *Angew. Chem.* **2018**, *130*, 706–710.

S7. L. Yu, J.F. Yang, B.Y. Guan, Y. Lu, X.W.D. Lou, Hierarchical Hollow Nanoprisms Based on Ultrathin Ni-Fe Layered Double Hydroxide Nanosheets with Enhanced Electrocatalytic Activity towards Oxygen Evolution, *Angew. Chem.* **2018**, *130*, 178–182.
S8. J. Liu, Y. Ji, J. Nai, X. Niu, Y. Luo, L. Guo, S. Yang, Ultrathin Amorphous Cobalt-Vanadium Hydr(oxy)oxide Catalysts for the Oxygen Evolution Reaction, *Energy Environ. Sci.* **2018**, in press.
S9. M. Xing, L.-B. Kong, M.-C. Liu, L.-Y. Liu, L. Kang, Y.-C. Luo, Cobalt Vanadate as Highly Active, Stable, Noble Metal-Free Oxygen Evolution Electrocatalyst, *J. Mater. Chem. A* **2014**, *2*, 18435–18443.
S10. X. Peng, L. Wang, L. Hu, Y. Li, B. Gao, H. Song, C. Huang, X. Zhang, J. Fu, K. Huo, P.K. Chu, In Situ Segregation of Cobalt Nanoparticles on VN Nanosheets via Nitriding of $Co_2V_2O_7$ Nanosheets as Efficient Oxygen Evolution Reaction Electrocatalysts, *Nano Energy* **2017**, *34*, 1–7.
S11. T. Gao, Z. Jin, M. Liao, J. Xiao, H. Yuan, D. Xiao, A Trimetallic V-Co-Fe Oxide Nanoparticles as an Efficient and Stable Electrocatalyst for Oxygen Evolution Reaction, *J. Mater. Chem. A* **2015**, *3*, 17763–17770.
